# Genome-wide identification of microRNA-related variants associated with risk of Alzheimer’s disease

**DOI:** 10.1038/srep28387

**Published:** 2016-06-22

**Authors:** Mohsen Ghanbari, M. Arfan Ikram, Hans W. J. de Looper, Albert Hofman, Stefan J. Erkeland, Oscar H. Franco, Abbas Dehghan

**Affiliations:** 1 Department of Epidemiology, Erasmus University Medical Center, 3000 CA Rotterdam, the Netherlands; 2 Department of Genetics, School of Medicine, Mashhad University of Medical Sciences, Mashhad, Iran; 3 Department of Neurology, Erasmus University Medical Center, 3000 CA Rotterdam, the Netherlands; 4 Department of Radiology, Erasmus University Medical Center, 3000 CA Rotterdam, the Netherlands; 5 Department of Hematology, Erasmus University Medical Center, 3000 CA Rotterdam, the Netherlands; 6 Department of Epidemiology, Harvard T.H. Chan School of Public Health, Boston, Mass, USA; 7 Department of Immunology, Erasmus University Medical Center, 3015 CN Rotterdam, the Netherlands

## Abstract

MicroRNAs (miRNAs) serve as key post-transcriptional regulators of gene expression. Genetic variation in miRNAs and miRNA-binding sites may affect miRNA function and contribute to disease risk. Here, we investigated the extent to which variants within miRNA-related sequences could constitute a part of the functional variants involved in developing Alzheimer’s disease (AD), using the largest available genome-wide association study of AD. First, among 237 variants in miRNAs, we found rs2291418 in the miR-1229 precursor to be significantly associated with AD (*p*-value = 6.8 × 10^−5^, OR = 1.2). Our *in-silico* analysis and *in-vitro* miRNA expression experiments demonstrated that the variant’s mutant allele enhances the production of miR-1229-3p. Next, we found miR-1229-3p target genes that are associated with AD and might mediate the miRNA function. We demonstrated that miR-1229-3p directly controls the expression of its top AD-associated target gene (*SORL1*) using luciferase reporter assays. Additionally, we showed that miR-1229-3p and *SORL1* are both expressed in the human brain. Second, among 42,855 variants in miRNA-binding sites, we identified 10 variants (in the 3′ UTR of 9 genes) that are significantly associated with AD, including rs6857 that increases the miR-320e-mediated regulation of *PVRL2.* Collectively, this study shows that miRNA-related variants are associated with AD and suggests miRNA-dependent regulation of several AD genes.

Alzheimer’s disease (AD) is the most common form of age-related neurodegenerative diseases worldwide manifested by the progressive loss of memory and cognitive decline^1^. A number of cellular and molecular mechanisms lead to AD occurrence^2^,^3^,^4^. Over the past few decades, enormous efforts have been made to discover risk factors that play a role in development of AD and new biomarkers that help in early diagnosis of AD^5^,^6^. Recently, microRNAs (miRNAs), a class of small non-coding RNAs with 20–24 nucleotides (nt) long, have gained widespread attention as important modulators of different biological processes. They have been shown to be involved in biological and pathological processes in the brain^7^,^8^,^9^. In addition, a number of dysregulated miRNAs have been reported to be associated with AD and are suggested as potential diagnostic biomarkers for AD^10^,^11^,^12^. MiRNAs repress translation or decrease the stability of target messenger RNAs (mRNAs), by binding of the nucleotides 2–8 from their 5′ end (known as the seed region) to complementary sequences in the 3′ UTR regions of target mRNAs^13^. MiRNAs are predicted to regulate the translation of up to 60% of all protein coding genes, indicating the involvement of miRNAs in most cellular processes^14^. Genetic variants in miRNA-encoding sequences can affect miRNA biogenesis and function^15^,^16^. Furthermore, variants that are located in the seed-matching regions of target genes may interfere with the interaction between miRNAs and their target genes, resulting in an altered expression level of the target transcript^17^,^18^. More recently, we and others have been able to show a number of variants in genomic sequences encoding miRNAs or in the 3′ UTRs of miRNA target genes that contribute to phenotypic variations and disease risk^18^,^19^,^20^,^21^,^22^. However, the association of such variants with the risk of AD has not yet been systematically investigated.

In the present study, we hypothesized that miRNA-related variants could constitute a part of the functional genomic variants influencing the risk of AD. To test this hypothesis, we investigated the association of all genetic variants located in miRNA genes as well as miRNA-binding sites in the 3′ UTR of their target genes with risk of AD using data from the largest available GWAS on late-onset AD^23^. We subsequently integrated our results with publicly available biological databases (such as miRNA and gene expression profiles) and performed experimental studies to provide evidence for the function of the identified variants in AD.

## Results

**Figure 1:**
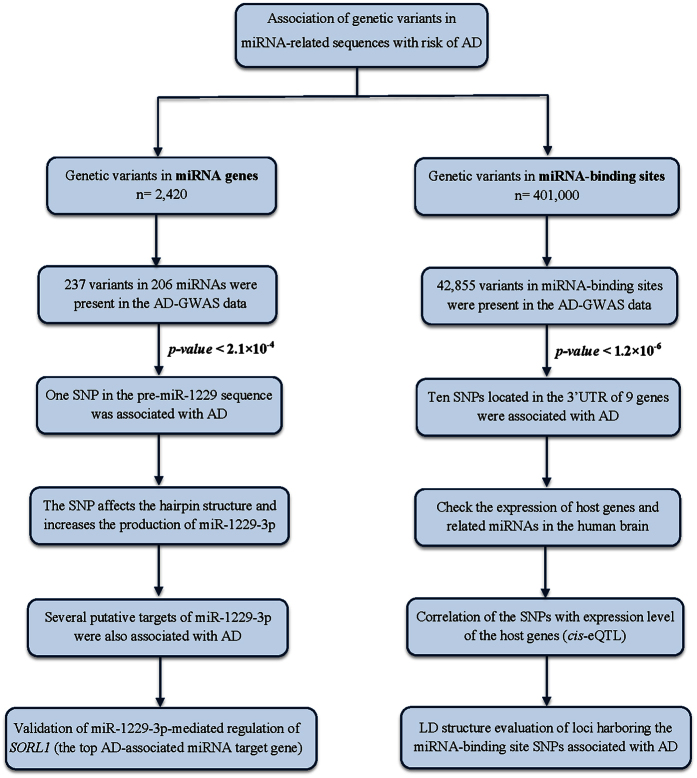
Identification of variants in miRNAs and miRNA-binging sites associated with Alzheimer’s disease. This flow chart shows our approach to detect variants that are located in miRNA-related sequences and are associated with AD. GWAS, genome-wide association studies; LD, linkage disequilibrium.

A flow chart of our approach to detect genetic variants within miRNAs and miRNA-binding sites that are associated with AD is shown in Fig. 1.

### Association of genetic variants in miRNAs with AD

#### A polymorphism in miR-1229 was associated with AD

Out of 237 variants located in 206 miRNAs, we found rs2291418 (Chr5:179225324, A > G), a low-frequency variant (MAF = 0.02) in the pre-miR-1229 sequence, to be significantly associated with the risk of AD (*p*-value = 6.8 × 10^−5^ and OR = 1.2). Supplementary Fig. S1 shows the association of rs2291418 and other variants within the related locus with AD. The associations of all miRNA-variants that are nominally associated (at p-value < 0.05) with the risk of AD are shown in Supplementary Table S1.

#### The impact of rs2291418 on miR-1229 structure and expression

**Figure 2:**
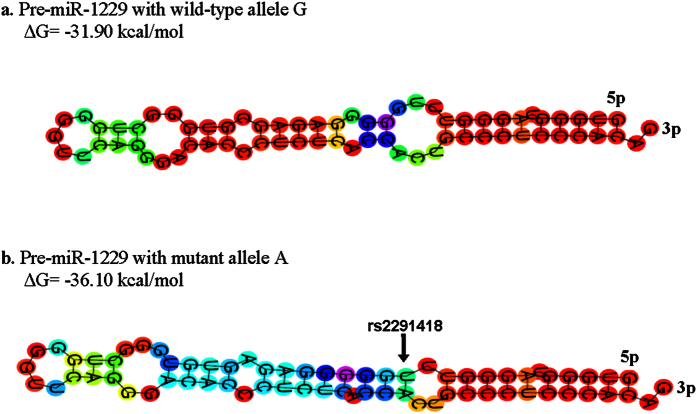
Schematic view of the RNAfold predicted secondary structures of the pre-miR-1229 containing either the wild type or the mutant allele. (**a**) RNAfold predicted hairpin structure of the pre-miR-1229 with wild type allele and the minimum free energy (MFE) of the thermodynamic ensemble (ΔG). (**b**) RNAfold predicted hairpin structure of the pre-miR-1229 with the mutant allele. The pre-miRNA structure with lower MFE is expected to thermodynamically be more stable. The SNP position is shown by arrow.

**Figure 3:**
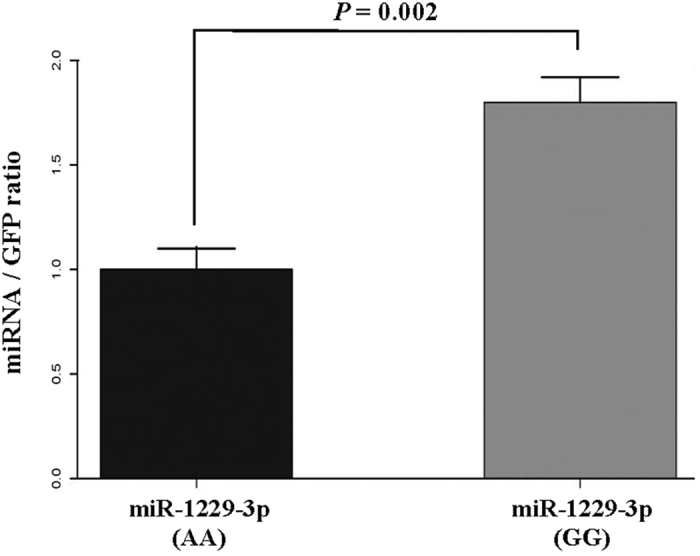
The effect of rs2291418 on the expression level of miR-1229-3p. This figure illustrates a significant increase in the level of mature miR-1229-3p from the mutant allele A compared to the wild type allele G relative to GFP (*p*-value = 0.002).

We generated the pre-miR-1229 hairpin structures containing the rs2291418 wild-type (G) and mutant (A) allele using the Vienna RNAfold algorithm^24^. We observed a −4.2 kcal/mol difference in the minimum free energy of the thermodynamic ensemble of the predicted structure of pre-miR-1229 mutant compared to wild type (Fig. 2). This analysis indicated that the variant’s mutant allele may enhance the stability of the pre-miR-1229 hairpin, suggesting an improvement of the pre-miRNA processing into the mature miR-1229. To experimentally show the impact of rs2291418 on the pre-miR-1229 processing, we examined the expression levels of mature miR-1229 with the wild type and mutant pre-miRNA sequences. To this end, HEK293 cells were transfected with pMCSV vectors expressing transcripts with EGFP and wild type or mutant pre-miR-1229. Both the pre-miR-1229 wild type and pre-miR-1229 mutant transfected cells had equal levels of the EGFP-miRNA transcripts. However, the mature miR-1229-3p levels were significantly increased by 70% in cells transfected with pre-miR-1229 containing the mutant allele compared to the wild-type allele (*p-*value = 0.002) (Fig. 3), indicating that the variant improves the processing or stability of miR-1229.

#### Multiple miR-1229-3p target genes were associated with AD

We assessed which target genes of miR-1229-3p are implicated in pathways in nervous system or neurological disorders and may have a role in developing AD. To this end, we compiled a list of all predicted target genes of miR-1229-3p using the two most commonly used miRNA target prediction algorithms (TargetScan and miRanda) (n = 960). Both miRNA and its target genes should be expressed in the same tissue for any biological function to be exerted. Thus, we checked the expression of miR-1229-3p in the brain. Our data showed that miR-1229-3p is abundantly expressed (average Ct-value: 28.8) in both white and gray matter of the human brain (Supplementary material, Table S2). In addition, miRNA expression databases (HMED and miRmine) show that miR-1229-3p is expressed at higher levels in the brain compared to other tissues, indicating an active regulatory role of miR-1229-3p on its target genes in the brain. Subsequently, we found that 750 miR-1229-3p target genes are expressed in the brain, using the Human Body Map 2.0 data.

We then used the AD-GWAS data in a candidate gene approach to identify those target genes that are likely to be involved in developing AD^23^. We assessed the association of genetic variants in the 750 brain-expressed target genes of miR-1229-3p with AD. Supplementary Table S3 shows ten target genes of miR-1229-3p with the most significant association with AD. In addition, our pathway analysis using IPA demonstrated several target genes of miR-1229-3p to be involved in the network of Nervous System Development and Neurological disease (Supplementary material, Table S4).

#### miR-1229-3p-mediated regulation of SORL1

**Figure 4:**
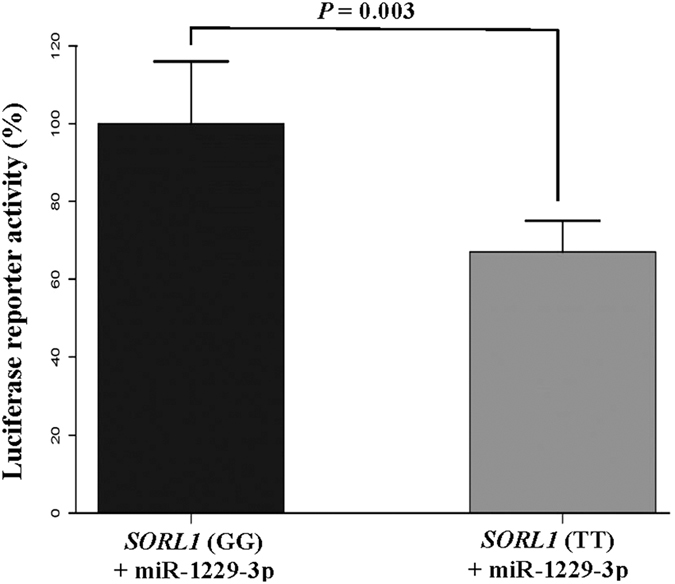
miR-1229-3p-mediated regulation of *SORL1*. This figure illustrates a significant decrease of the mean relative luciferase activity of wild type *SORL1* reporter (TT), compared to the mutated *SORL1* reporter (GG) in the presence of miR-1229-3p (*p*-value = 0.003).

We selected *SORL1*, which is the most significant AD-associated target gene of miR-1229-3p, and examined whether the miRNA controls its expression level *in vitro*. We generated expression vector containing the pre-miR-1229-3p sequence and co-transfected the construct with Luciferase reporters containing the 3′ UTR (wild-type or mutant miR-1229-3p binding site) of *SORL1*. We found that over-expression of miR-1229-3p significantly decreases the Luciferase activity of the reporter containing the wild type *SORL1* fragment, compared to the mutated *SORL1* reporter (*p*-value = 0.003) (Fig. 4). In addition, this experiment showed a dose-dependent regulation of *SORL1* by miR-1229-3p (Supplementary Fig. S2).The Human Body Map 2.0 data further show that *SORL1* is abundantly expressed in the brain (Supplementary material, Table S3). These data strongly suggest for miR-1229-3p-mediated regulation of *SORL1* in the human brain.

### Association of genetic variants in miRNA-binding sites with AD

#### Ten miRNA binding site variants were associated with AD

**Figure 5:**
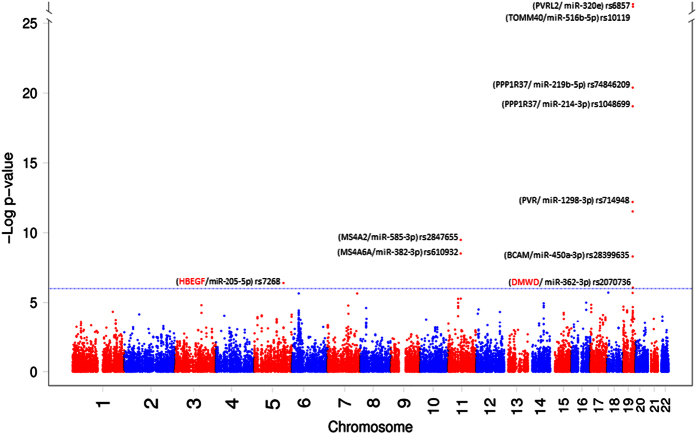
Association of miRNA-binding site variants with Alzheimer’s disease. The Manhattan plot shows the association of 42,855 SNPs in miRNA-binding sites with AD. Dashed line indicates the significant study threshold which was set at 1.2 × 10^−6^. Genes hosting the variants significantly associated with AD are highlighted. The two newly identified genes for AD are depicted in red.

We examined the association of 42,855 variants in putative miRNA-binding sites with AD that are illustrated in a Manhattan plot (Fig. 5). Of these, ten variants located in the 3′ UTR of 9 different genes passed the Bonferroni corrected significance threshold (*p*-value < 1.2 × 10^−6^). These variants would potentially interfere with miRNA-mediated regulation of their host genes by disrupting, creating or modifying a number of miRNA-binding sites that are depicted in Supplementary Table S5. Out of 9 genes hosting the identified variants, the association of 7 genes with AD have been reported in the original GWAS (*p*-value < 5 × 10^−8^)^23^. In addition, we found two new susceptibility genes to be potentially associated with AD, including *DMWD* (*p*-value = 8.48 × 10^−7^) and *HBEGF* (*p*-value = 3.96 × 10^−7^). Regional plots showing association of the ten miRNA-binding site SNPs and their close by variants with AD are shown in Supplementary Fig. S2.

#### Functional annotation of the identified miRNA-binding site variants

We searched several databases and web tools to provide further insight into the functional role of ten AD-associated SNPs in miRNA-binding sites. Supplementary Table S6 shows a list of all web tools and databases that we used for our analyses. First, we examined the cis-expression quantitative trait loci (eQTLs) for 9 genes hosting the identified SNPs. We found that rs6857, rs2070736, rs2847655, rs10119 and rs610932 are correlated with differential expression levels of their host genes in blood (Table 1), showing that these SNPs alter expression levels of their host genes. In addition, the cis-regulatory effects of rs610932 and rs714948 on the expression of *MS4A6A* and *PVR* in cerebellum and temporal cortex have been reported^25^. Using the HaploReg web tool (v4), we found that 7 of the identified SNPs have no non-synonymous proxy SNPs in their loci, which suggest the identified SNPs are functional variants (Supplementary material, Table S7). The Human Body Map 2.0 RNA-seq data show that 8 out of the 9 host genes are expressed in the human brain. The miRNA expression databases further show that miR-214-3p, miR-212-5p, miR-204-3p, miR-362-3p, miR-450a and miR-320 are expressed in the brain. A summary of our findings regarding on potential functionality of the ten identified variants in miRNA-binding sites are shown in Table 1 and Supplementary Table S7. These data suggest that the genes associated with AD containing variants in 3′ UTRs may be affected because of aberrant miRNA activities.

## Discussion

Recent studies have shown the critical role of miRNAs in neuronal development and function^19^,^26^,^27^,^28^. In addition, a number of miRNAs have been reported to be implicated in the development of AD^7^,^8^,^9^,^10^,^11^,^12^. However, these studies were mainly focused on differentially expressed miRNAs and target genes using expression arrays in a small number of samples. There are also a few studies that have linked miRNAs with neurodegenerative disorders using genetic association in a candidate gene approach^29^,^30^. In this study, we performed a genome-wide investigation to identify genetic variants in miRNAs and in miRNA-binding sites that are associated with AD using data from the thus far largest GWAS on AD^23^.

We found that rs2291418 in the miR-1229 precursor, a miRNA which is abundantly expressed in the brain, is associated with the risk of AD. The pre-miR-1229 is precursor of two mature miRNAs (miR-1229-3p and miR-1229-5p), however, the 3p miRNA has been shown to be the predominant product^31^. *In silico* analysis indicated that the mutant allele improves the stability of the hairpin structure of pre-miR-1229. Our miRNA expression experiments further showed that the mutant allele enhances the stability or processing of mature miR-1229-3p. It has been shown that variants located at the stem region of miRNA precursors could alter Drosha-mediated processing^22^,^32^,^33^. Although, many studies have shown that genetic variants within pre-miRNA sequences reduce the mature miRNA levels^22^,^33^,^34^, a few studies have reported such variants to enhance miRNA processing and expression^33^.

We revealed miR-1229-3p target genes that are associated with AD. An optimal approach would have been to check the co-expression of all potential target genes of miR-1229-3p in brain tissues and examine their correlation with the miRNA expression to distill the potential mediators. However, collecting samples carrying the rare mutant allele (MAF = 0.03) of miR-1229-3p is challenging and was not possible for the investigators. Alternatively, we used GWAS data to identify the target genes that are more likely to be involved in the development of AD. By this approach, we could highlight a number of miR-1229-3p target genes that are known for neurological-related pathways and diseases, including *SORL1*, *MCFD2*, *COL25A1* and *BMP2*^35^,^36^,^37^. This approach, however, may overlook certain target genes that mediate the miRNA function on AD. We experimentally verified that miR-1229-3p interacts with the predicted target site in the 3′ UTR of *SORL1* which is the most significant AD-associated miR-1229-3p target and is abundantly expressed in the human brain. These data indicate that miR-1229-3p directly controls the expression of *SORL1* in the brain. Several studies have reported decreased *SORL1* expression to be mechanistically involved in causing AD^37^. It has been shown that *SORL1* directs trafficking of the amyloid precursor protein (APP) into recycling pathways and that when *SORL1* is reduced, APP is sorted into amyloid beta peptide in AD^35^. Therefore, a higher expression level of miR-1229-3p in rs2291418 mutant allele carriers may reduce *SORL1* level and subsequently increase AD risk.

Furthermore, we identified ten variants in miRNA-binding sites that are associated with AD. Among them, rs6857 in *PVRL2* is most significantly associated with AD^38^,^39^. The rs6857 mutant allele is predicted to create a target site for miR-320e in the 3′ UTR of *PVRL2*. Previously, we have experimentally shown that the expression of the mutant *PVRL2* is regulated by miR-320e, resulting in a decreased level of the *PVRL2* transcript^18^. In agreement, the eQTL analysis showed a correlation between the rs6857 mutant allele and a lower expression level of *PVRL2*. Both *PVRL2* and miR-320e are expressed in the brain^40^. These data suggest that rs6857 increases the risk of AD, at least in part, through down-regulation of *PVRL2* by miR-320e.

We found that two SNPs in miRNA-binding sites of *MS4A6A* and *MS4A2* are associated with AD susceptibility. SNP rs610932 within the 3′ UTR of *MS4A6A* is predicted to disrupt a binding site of miR-382-3p. *MS4A6A* is a well-known gene for susceptibility to AD^25^,^41^,^42^ and miR-382-3p is expressed in the brain. We observed that carriers of the minor allele containing rs610932 SNP, have a higher expression level of *MS4A6A* transcript in the blood eQTL data. The variant has been also shown to be associated with an altered expression level of *MS4A6A* in cerebellum and temporal cortex^25^. Therefore, an allele-specific regulation of *MS4A6A* by miR-328-3p may explain part of the observed association with AD. We also identified two SNPs in the 3′ UTR of *PPP1R37* which can potentially disrupt binding sites of miR-219b-5p and miR-214-3p. *PPP1R37* gene has previously been reported to be associated with AD and is expressed in the brain^23^,^43^. The both miRNAs, have been suggested to be implicated in neuro-degenerative disorders including Parkinson’s and Huntington’s disease^44^. A disruption of miR-214-3p and miR-219b-5p mediated regulations of *PPP1R37* may be considered as a reason for the association of rs74846209 and rs1048699 with AD. Future experimental studies are needed to determine the function of these variants in AD.

We report *DMWD* and *HBEGF* as two new susceptibility genes for AD. First, the rs2070736 creates potential binding sites for miR-362-3p and miR-329-3p in the 3′ UTR of *DMWD*. These miRNAs and also *DMWD* are expressed in the brain^45^,^46^. The eQTL analysis further showed that the mutant allele is associated with lower expression level of the *DMWD* transcript, suggesting that rs2070736 change the expression levels of *DMWD* by affecting miRNA regulation. *DMWD* plays a role in developing mental symptoms in severe cases of myotonic dystrophy^47^. Interestingly, Alzheimer’s neurofibrillary changes in brain are reported to be present in myotonic dystrophy^48^. Together, the link between myotonic dystrophy and AD is notable and may indicate a potential role for *DMWD* in AD as well. Second, we found that rs7268 in the 3′ UTR of *HBEGF* is associated with AD and is expected to create a binding site for miR-205-5p, a brain-expressed miRNA. It has been reported that the membrane bound for *HBEGF* is constitutively expressed and present on the blood-brain barrier. *HBEGF* expression is also shown to be abundantly up-regulated in cerebral blood vessels in inflammatory situations such as AD, Parkinson’s disease, stroke, epilepsy and encephalitis^49^,^50^. Therefore, the aberrant miR-205-5p regulation of *HBEGF* could be a potential mechanism underlying the identified association.

In conclusion, in a genome-wide investigation, we identified a variant in miR-1229 and ten variants in miRNA-binding sites located in the gene 3′ UTRs that are associated with the risk of AD. Our in silico and *in vitro* analyses showed miR-1229-3p-mediated regulation of *SORL1*, which is a well-known gene involved in AD. Further experimental studies on the identified variants as well as miRNA profiling in AD patients are needed to determine the role of highlighted miRNAs and target genes in pathogenesis of AD. Finally, our study suggest altered miRNA-dependent regulation of genes due to variants in 3′ UTRs may explain part of the associations identified by GWAS.

## Methods

### Identification of genetic variants in miRNAs and miRNA-binding sites

We made a dataset of all genetic variants that are located in miRNA-related sequences using two online databases: miRNASNP and PolymiRTS^51^,^52^. The mature miRNAs of 20–24 nt in length are produced by processing of the precursor miRNAs (pre-miRNA) (60–80 nt)^13^. We thus investigated all variants that are located in human precursor and mature miRNA sequences. We retrieved a total of 2,420 variants in all miRNA-encoding sequences. We excluded variants with minor allele frequency (MAF) < 0.01. Of these, we included 237 single-nucleotide polymorphisms (SNPs) in 206 different miRNAs that were present in the recent GWAS of AD^23^. In addition, we retrieved almost 401,000 variants in the 3′ UTR of all human miRNA target genes that are predicted to affect the match to the seed region of miRNAs. Of these, we included 42,855 SNPs with MAF > 0.01 and present in the GWAS of AD^23^.

### Genome-wide association study on AD

To examine the association of variants in miRNA-related sequences with AD, we used the summary statistics data (stage 1) from the thus far largest GWAS on late-onset AD, including data from 17,008 AD cases and 37,154 controls on 7,055,881 SNPs, was reported by IGAP consortium^23^. A description of the consortium data sets and participants are described elsewhere^23^. We adjusted *p*-value using Bonferroni correction for the number of tests and significant threshold was set at 2.1 × 10^−4^ (0.05/237) for SNPs in miRNAs and 1.2 × 10^−6^ (0.05/42,855) for SNPs in miRNA-binding sites.

### Prediction of the variant effect on miRNA structure

When a miRNA-variant was associated with AD, we used the Vienna RNAfold algorithm (ViennaRNA package 2.0) to predict the effect of that variant on the secondary structure of the pre-miRNA (hairpin structure)^24^. The differences in minimum free energy (MFE) of the thermodynamic ensemble of pre-miRNA sequences containing the mutant versus the wild type alleles may affect the pre-miRNA that is processed to form mature miRNA.

### Impact of variant on miRNA expression

We cloned the pre-miR-1229 sequence containing either the wild type or mutant allele behind the gene encoding green fluorescent protein (GFP) in the expression plasmid MSCV-BC (Murine Stem Cell Virus-Bar Coded)^53^, resulting in GFP-miRNA fusion transcripts. The inserts of all constructs were validated by Sanger sequencing. HEK293 cell transfection, total RNA isolation and quantitative PCRs were performed as previously described^53^. All primers are shown in Supplementary Table S8. The experiment was performed in triplicate.

### Association of miR-1229-3p target genes with AD

We compiled a list of all predicted target genes of the identified miRNA using two online databases, TargetScan v7.0 (http://www.targetscan.org)^54^ and miRanda (http://www.microrna.org/microrna/home.do)^55^. The miRNA target genes that are listed in both databases were used for our analyses. We then examined which of the miRNA putative targets are expressed in the human brain using the Human Body Map 2.0 RNA-seq data. Subsequently, we used the AD-GWAS data in a candidate gene approach to identify those target genes that are likely to be involved in AD. We retrieved the summary statistics for the association of all genetic variants in the target genes with AD from the GWAS data. The significance threshold was set using the Bonferroni correction based on the number of studied SNPs. Additionally, we explored whether target genes of the identified miRNA may play a role in neurologic-related pathways. To do this, we used Ingenuity Pathway Analysis (IPA) (http://www.ingenuity.com/products/ipa/), a knowledge database generated from peer-reviewed scientific publications that enables the discovery of highly represented biological mechanisms, pathways or functions most relevant to the genes of interest from large, quantitative datasets. We uploaded all miRNA target genes and performed a core IPA analysis with default settings. We mapped the miRNA target genes to biological functions or canonical pathways to see whether they are enriched in neurologic-related networks.

### Luciferase reporter assay

Primers were designed to amplify the 3′ UTR sequence of target gene included in the restriction enzyme sites XbaI for the forward primer and ApaI for the reverse. The SORL1 3′ UTR sequences (wild-type and mutated), containing the putative binding site of miR-1229-3p, were amplified and cloned into the pGL3 luciferase reporter vector downstream of the Luciferase open reading frame (Promega). The primers are shown in Supplementary Table S9. Similarly, pre-miR-1229 sequence was amplified using a forward primer containing a XhoI restriction site and a reverse primer containing a EcoRI restriction site. The amplified miRNA sequence was then cloned into the pMSCV-BC vector as previously described^53^. The inserts of all constructs were validated by Sanger sequencing. HEK293 cells were plated into 96-well plates and co-transfected with MSCV-miR-1229, pGL3 vector containing the SORL1 3′ UTR and a plasmid expressing the Renilla transfection control. Luciferase activity was determined with the Dual-Glo Luciferase Assay System according to manufacturer’s protocol (Promega). Renilla luciferase activity was used for normalization. All experiments were performed in triplicate.

### Expression of the identified miRNAs and target genes in relevant tissues

We examined the expression of miR-1229-3p in the human brain. The brain tissues (3 gray matter and 3 white matter) were obtained from the Netherlands Brain Bank (Amsterdam, The Netherlands). All samples were free of neurological disease. For isolation of total RNA, five cryopreserved sections of 40 μm were homogenized in 750 μl Trizol LS reagent (Invitrogen, Carlsbad, CA, USA). Total RNA was isolated from 6 brain samples and. the concentration and purity of RNA samples were determined with a NanoDrop ND-1000 spectrophotometer (NanoDrop, Wilmington, DE). The expression level of miR-1229-3p was determined with TaqMan MicroRNA Assay according to manufacturer’s protocol (Applied Biosystems, Foster City, CA, USA). RNU6B was used as an endogenous control. All experiments were performed in triplicate.

The Human Body Map 2.0 RNA-seq data (http://www.ensembl.info/blog/2011/05/24/human-bodymap-2-0-data-from-illumina/) was used to examine the expression of miRNA target genes across different tissues. To scan the expression of other miRNAs in the brain, we used HMED (http://bioinfo.life.hust.edu.cn/smallRNA/index.php) and miRmine (http://guanlab.ccmb.med.umich.edu/mirmine/help.html) databases. Moreover, we used human miRNA expression data from four independent studies showing miRNAs that are differentially expressed (both up- and down-regulated) in different brain tissues and also AD patients^40^,^46^,^56^,^57^.

### Functional characteristics of the identified miRNA-binding site variants

The list of variants in miRNA-binding sites associated with AD submitted to the SNAP web tool (http://www.broadinstitute.org/mpg/snap/id) using R^2^ threshold >0.8, limit distance 500 kb, and population panel CEU to retrieve their proxy SNPs in the 1000 Genomes project. We used the HaploReg (v4) web tool to predicts the effect of these SNPs on protein structure, gene regulation, and splicing. This analysis helps us to know whether there are other variants in high LD with the identified SNPs that may drive the observed association. We scanned the expression quantitative trait loci (cis-eQTL) data using GTEx (http://www.broadinstitute.org/gtex/) and HaploReg (4.1) that show the correlation of the variants with the expression of nearby genes in several tissues^58^,^59^. Other information, including miRNA sequences, host gene, miRNAs family and cluster amongst others, and miRNA conservation in different species was obtained from miRBase (release 20)^60^ and TargetScan (v7.0) databases.

## Additional Information

**How to cite this article**: Ghanbari, M. *et al*. Genome-wide identification of microRNA-related variants associated with risk of Alzheimer’s disease. *Sci. Rep.* **6**, 28387; doi: 10.1038/srep28387 (2016).

## Supplementary Material

Supplementary Information

## Figures and Tables

**Table 1:** A summary of supporting evidence for the potential functionality of the 10 identified miRNA-binding site variants associated with Alzheimer’s disease.

		rs6857	rs2847655	rs610932	rs74846209	rs1048699	rs10119	rs714948	rs28399635	rs2070736	rs7268
GWAS	*p*-value	2.5 × 10^−575^	3.3 × 10^−10^	3.1 × 10^−9^	4.1 × 10^−21^	9.0 × 10^−20^	1.2 × 10^−342^	6.3 × 10^−13^	5.0 × 10^−9^	8.5 × 10^−7^	4.0 × 10^−7^
	OR	3.2	1.1	0.9	2.3	1.2	1.3	1.1	0.9	1.1	1.1
Gene	Host gene	PVRL2	MS4A2	MS4A6A	PPP1R37	PPP1R37	TOMM40	PVR	BCAM	DMWD	HBEGF
	Brain Exp.	10.0	NA	6.2	11.0	11.0	9.7	5.7	3.0	11.5	9.2
miRBS	miRNA ID	miR-320e	miR-585-3p	miR-382-3p	miR-219b-5p	miR-214-3p	miR-516b-5p	miR-1298-3p	miR-450a-3p	miR-362-3p	miR-205-5p
	Score change	−0.19	−0.48	−0.10	−0.29	−0.08	−0.06	−0.08	−0.16	−0.15	−0.12
eQTL	Blood	9.5 × 10^−5^(−)	6.5 × 10^−22^(−)	2.1 × 10^−35^(+)	−	−	+	−	−	2.4 × 10^−10^ (+)	−
	Brain	−	−	+	−	−	−	+	−	−	−
Proxy	Nr. proxies	0	94	72	18	18	0	1	0	18	8
	Non-synon	0	0	1	1	1	0	0	0	0	0

miRBS; miRNA binding site, OR; Odds ratio, Exp; Expression, Non-synon; Non-synonymous proxy SNP, NA; Not available. miRNAs in this table are highly conserved miRNAs related to the SNPs. Score change; Context score change using PolymiRTS database (v3.0).
