# Supplementary material for: Genome-wide identification of microRNA-related variants associated with risk of Alzheimer’s disease
Source: Sci Rep. 2016 Jun 22;6:28387. doi: 10.1038/srep28387 (PMC4916596; doi:10.1038/srep28387)
Supplement: Supplementary Information [file srep28387-s1.doc]

**Genome-wide identification of microRNA-related variants associated with risk of Alzheimer’s disease**

Mohsen Ghanbari^1,2,*^, M. Arfan Ikram^1,3,4^, Hans W.J. de Looper^5^, Albert Hofman^1,6^, Stefan J. Erkeland^7^, Oscar H. Franco^1^, Abbas Dehghan^1,*^

1. Department of Epidemiology, Erasmus University Medical Center, 3000 CA Rotterdam, the Netherlands.

2. Department of Genetics, School of Medicine, Mashhad University of Medical Sciences, Mashhad, Iran.

3. Department of Neurology, Erasmus University Medical Center, 3000 CA Rotterdam, the Netherlands.

4. Department of Radiology, Erasmus University Medical Center, 3000 CA Rotterdam, the Netherlands.

5. Department of Hematology, Erasmus University Medical Center, 3000 CA Rotterdam, the Netherlands.

6. Department of Epidemiology, Harvard T.H. Chan School of Public Health, Boston, Mass, USA

7. Department of Immunology, Erasmus University Medical Center, 3000 CA Rotterdam, the Netherlands.

*** Corresponding authors:**

1- Mohsen Ghanbari, MD MSc

Email: [m.ghanbari@erasmusmc.nl](mailto:m.ghanbari@erasmusmc.nl)

2- Abbas Dehghan, MD PhD

Email: a.dehghan@erasmusmc.nl

Department of Epidemiology

Erasmus Medical Center

Wytemaweg 80, 3015CN Rotterdam, Rotterdam, The Netherlands

Phone: 0031 10 70 38955

Fax: 0031 10 70 44657

**Supplementary information**

**Table S1.** miRNA-SNPs that are nominally associated (p < 0.05) with Alzheimer’s disease

**Table S2.** Expression of miR1229-3p in the human brain samples

**Table S3.** miR-1229-3p target genes with the most significant association with Alzheimer’s disease

**Table S4.** Ingenuity pathway analysis (IPA) for 750 target genes of miR-1229-3p

**Table S5.** Ten miRNA-binding site variants associated with Alzheimer’s disease

**Table S6.** List of web tools and databases that we used to identify the association between miRNA-related variants and Alzheimer’s disease

**Table S7.** Functional annotation of the 10 identified miRNA binding site variants associated with Alzheimer’s disease

**Table S8.** List of the primers for the cloning of the miR-1229 precursor containing wild-type and mutant alleles

**Table S9.** List of the primers for the cloning of the wild type and mutant 3’UTR of *SORL1*

**Figure S1.** Regional plot showing the association of miR-1229 variant with Alzheimer’s disease

**Figure S2.** A dose-dependent regulation of *SORL1* expression by miR-1229-3p

**Figure S3.** Regional association plots of the identified miRNA-binding site variants associated with Alzheimer’s disease

**Table S1. miRNA-SNPs that are nominally associated (p < 0.05) with Alzheimer’s disease**

| **SNP ID** | **Chr.** | **Position** | **A1/A2** | **Effect** | **SE** | ***p*-value** | **miRNA ID** | **Location**  **in miRNA** |
| --- | --- | --- | --- | --- | --- | --- | --- | --- |
| rs2291418 | 5 | 179798324 | G/A | 0.1756 | 0.0441 | **6.9E-05** | mir-1229 | Mature |
| rs2292181 | 3 | 44903434 | G/C | -0.1293 | 0.0366 | 4.0E-04 | mir-564 | Pre-miR |
| rs10423365 | 19 | 47212593 | A/G | -0.0435 | 0.0157 | 0.005 | mir-320e | Pre-miR |
| rs138079376 | 1 | 201719605 | G/A | 0.2789 | 0.1042 | 0.007 | mir-5191 | Pre-miR |
| rs745666 | 17 | 72744798 | G/C | -0.056 | 0.0215 | 0.009 | mir-3615 | Pre-miR |
| rs35196866 | 9 | 134379472 | C/A | 0.055 | 0.0217 | 0.011 | mir-4669 | Pre-miR |
| rs17759989 | 17 | 61021611 | A/G | 0.1218 | 0.0482 | 0.011 | mir-633 | Pre-miR |
| rs2663345 | 17 | 925764 | A/G | -0.0547 | 0.0226 | 0.016 | mir-3183 | Pre-miR |
| rs12451747 | 17 | 12820632 | A/C | -0.0382 | 0.0158 | 0.016 | mir-1269b | Pre-miR |
| rs73147065 | 20 | 62574006 | A/C | -0.0632 | 0.0263 | 0.017 | mir-647 | Pre-miR |
| rs6505162 | 17 | 28444183 | A/C | 0.0379 | 0.016 | 0.018 | mir-423 | Pre-miR |
| rs74469188 | 16 | 81644970 | T/C | 0.0598 | 0.0254 | 0.019 | mir-6504 | Pre-miR |
| rs4112253 | 19 | 54786022 | G/C | -0.0494 | 0.0212 | 0.020 | mir-4752 | Pre-miR |
| rs116814212 | 6 | 32717702 | NA | -0.0379 | 0.0169 | 0.025 | mir-3135b | Pre-miR |
| rs7235219 | 18 | 46196998 | A/G | -0.1859 | 0.0882 | 0.035 | mir-4743 | Pre-miR |
| rs8667 | 19 | 50436371 | G/A | -0.0336 | 0.0163 | 0.040 | mir-4751 | Pre-miR |
| rs76800617 | 17 | 8090294 | A/G | 0.1215 | 0.0594 | 0.041 | mir-4521 | Pre-miR |
| rs897984 | 16 | 30886643 | T/C | -0.0323 | 0.0161 | 0.045 | mir-4519 | Pre-miR |
| rs2292832 | 2 | 240456086 | T/C | -0.0402 | 0.0201 | 0.046 | mir-149 | Pre-miR |
| rs73235381 | 8 | 26906402 | C/T | -0.0998 | 0.0504 | 0.048 | mir-548h-4 | Pre-miR |

Chr., Chromosome; A1, Allele 1; A2, Allele 2; SE, Standard error; Pre-miR, Precursor miRNA; Mature, Mature miRNA sequence.

**Table S2. Expression of miR1229-3p in the human brain samples**

| **Sample ID** | **Ct value**  **U6 control** | **Ct value**  **miR-1229-3p** | **dCt**  **(Ct miR- Ct U6)** |
| --- | --- | --- | --- |
| Gray matter sample 1 | 21.1 | 29.6 | 8.4 |
| Gray matter sample 2 | 21.7 | 29.5 | 7.8 |
| Gray matter sample 3 | 21.1 | 28.6 | 7.5 |
| White matter sample 1 | 20.4 | 28.3 | 7.9 |
| White matter sample 2 | 19.0 | 27.9 | 8.9 |
| White matter sample 3 | 21.1 | 28.9 | 7.8 |

U6 snRNA, an endogenous control for miRNA expression study; dCt, delta Ct value.

**Table S3. miR-1229-3p target genes with the most significant association with Alzheimer’s disease**

| **Gene** | **SNP ID** | **P-value** | **Gene name** | **Expression in the brain** |
| --- | --- | --- | --- | --- |
| *SORL1 | rs11218343 | 4.98E-11 | Sortilin-Related Receptor, L(DLR Class) A Repeats Containing | 26.94 |
| PFDN1 | rs6580473 | 8.73E-06 | Prefoldin Subunit 1 | 54.75 |
| *MCFD2 | rs6715234 | 8.82E-06 | Neural Stem Cell-Derived Neuronal Survival Protein | 34.90 |
| RAB31 | rs1015228 | 1.20E-05 | Ras-Related Protein Rab-22B | 61.99 |
| LRRC32 | rs1893306 | 3.35E-05 | Leucine Rich Repeat Containing 32. | 10.29 |
| *COL25A1 | rs11736110 | 3.63E-05 | Alzheimer Disease Amyloid-Associated Protein | 1.12 |
| ZNF594 | rs8081019 | 3.72E-05 | Zinc Finger Protein 594 | 1.02 |
| JARID2 | rs764650 | 5.90E-05 | Jumonji, AT Rich Interactive Domain 2 | 4.10 |
| *BMP2 | rs6054767 | 5.92E-05 | Bone Morphogenetic Protein 2 | 2.56 |
| RFX3 | rs10972598 | 6.59E-05 | Regulatory Factor X 3 | 4.60 |

The *p*-value for each target gene is *p*-value of the SNP in the gene with the most significant association in GWAS of AD (Lambert, et al., 2013). The expression values are as fragments per kb of exon per million reads (FPKMs), which is a measure of gene expression normalized to size of the gene and RNA-seq library size in the Human Body Map 2.0 data. *Genes that have been shown to be involved in neurological-related pathways (GeneCards database).

**Table S4. Ingenuity pathway analysis (IPA) for 750 target genes of miR-1229-3p**

| **miR-1229-3p target genes** | | |
| --- | --- | --- |
| **Top Associated Network** | | |
| Gene Expression, Tissue Development, Cellular Development  Cell Morphology, Renal and Urological System Development and Function, Cellular Development  Psychological Disorders, Cellular Function and Maintenance, Molecular Transport  Hematological System Development and Function, Humoral Immune Response  Nervous System Development and Function | | |
| **Top Canonical Pathways**  **Name p-value # Molecules** | | |
| Circadian Rhythm Signaling  GDNF Family Ligand-Receptor Interactions Parkinson's Signaling  Ephrin A Signaling  IGF-1 Signaling | 4.98E-03  5.76E-03  1.20E-02  1.41E-02  1.94E-02 | 9.1 % 3/33  5.9 % 4/68  12.5 % 2/16  6.2 % 3/48  4.1 % 4/97 |
| **Physiological System Development and Function**  **Name** | **p-value** | **# Molecules** |
| Nervous System Development and Function  Organ Morphology  Organismal Development  Cell-mediated Immune Response  Hematological System Development and Function | 2.10E-02 - 1.29E-05  2.10E-02 - 1.29E-05  2.10E-02 - 1.29E-05  7.52E-03 - 2.18E-05  2.03E-02 - 2.18E-05 | 50  23  53  20  28 |

The p-values are calculated using the right-tailed Fisher Exact Test and a p-value less than 0.05 indicates a statistically significant, non-random association.

**Table S5. Ten miRNA-binding site variants associated with Alzheimer’s disease**

| **SNP ID** | **Position** | **MAF** | **Gene ID** | **A1** | **Putative miRNA binding sites**  **in presence of A1 allele** | **A2** | **Putative miRNA binding sites**  **in presence of A2 allele** |
| --- | --- | --- | --- | --- | --- | --- | --- |
| rs28399635 | 45323243 | 0.28 | *BCAM* | G | miR-450a-2-3p|miR-2110|miR-3150a-3p|  miR-6763-5p|miR-6810-5p|miR-6857-5p | A | miR-661|miR-4667-5p|miR-4700-5p|miR-6735-3p|miR-6852-5p|miR-6878|miR-8089 |
| rs2070736 | 46286714 | 0.24 | *DMWD* | C | None | T | miR-329-3p|miR-362-3p |
| rs7268 | 140332965 | 0.47 | *HBEGF* | A | None | T | miR-205-5p|miR-4724-3p |
| rs2847655 | 59865671 | 0.23 | *MS4A2* | C | miR-3945|miR-4253|miR-6862-5p | T | miR-585-3p|miR-1268a|miR-1268b |
| rs610932 | 59939307 | 0.45 | *MS4A6A* | A | miR-382-3p|miR-3120-5p|miR-4743-3p|  miR-4778-5p|miR-6888-3p | C | miR-626|miR-6876-3p |
| rs1048699 | 45650386 | 0.08 | *PPP1R37* | T | miR-214-3p|miR-761|miR-3619-5p|  miR-4520a-3p|miR-6510-5p | C | None |
| rs74846209 | 45650099 | 0.08 | *PPP1R37* | T | miR-212-5p|miR-219b-5p|miR-3189-3p | G | miR-890|miR-3171 |
| rs714948 | 45165912 | 0.03 | *PVR* | A | miR-204-3p|miR-619-5p|miR-1298-3p|miR-3192-5p|miR-4314|miR-4646-5p|miR-6506 | C | miR-432-5p|miR-1289|miR-3198|  miR-4294|miR-4309 |
| rs6857 | 45392254 | 0.11 | *PVRL2* | T | miR-645|miR-3929|miR-4419b|  miR-4478|miR-4505|miR-5787 | C | miR-320e |
| rs10119 | 45406673 | 0.25 | *TOMM40* | A | None | G | miR-516b-5p|miR-1299|miR-4782-5p|  miR-5706|miR-6128|miR-875-3p |

MAF, Minor allele frequency; A1, Wild type allele, A2; Mutant allele.

**Table S6. List of web tools and databases that used to identify the association between miRNA-related SNPs and Alzheimer’s disease**

| **Name** | **Main feature** | **URL** | **Reference** |
| --- | --- | --- | --- |
| **miRNASNP v2** | SNPs in miRNA-related sequences | <http://bioinfo.life.hust.edu.cn/miRNASNP2/index.php> | 51 |
| **PolymiRTS 3.0** | SNPs in miRNA binding sites | [http://compbio.uthsc.edu/miRSNP](http://compbio.uthsc.edu/miRSNP/) | 52 |
| **IGAP** | International Genomics of Alzheimer's Project provides GWAS data | <http://web.pasteur-lille.fr/en/recherche/u744/igap/igap_download.php> | 24 |
| **Vienna RNA** | Prediction of SNP effect on miRNA secondary structure | [http://rna.tbi.univie.ac.at](http://rna.tbi.univie.ac.at/) | 23 |
| **TargetScan 7.0** | miRNA target prediction | [http://www.targetscan.org](http://www.targetscan.org/) | 54 |
| **MiRanda** | miRNA target prediction | <http://www.microrna.org/microrna/home.do> | 55 |
| **IPA** | A comprehensive software on biological and pathway analysis | <http://www.ingenuity.com/products/ipa> | - |
| **Human BodyMap** | RNASeq data from Illumina’s Human Body Map 2.0 project | [http://www.ensembl.info/blog/2011/05/24/human-bodymap-2-0-data-from-illumina](http://www.ensembl.info/blog/2011/05/24/human-bodymap-2-0-data-from-illumina/) | - |
| **HMED** | The human miRNA expression database | http://bioinfo.life.hust.edu.cn/smallRNA/index.php | - |
| **miRmine** | A Human miRNA Expression Database | http://guanlab.ccmb.med.umich.edu/mirmine/help.html | - |
| **SNAP** | Proxy SNPs based on linkage disequilibrium and physical distance | https://www.broadinstitute.org/mpg/snap/ldsearch.php | - |
| **HaploReg v4** | For exploring annotations of variants on haplotype blocks | http://www.broadinstitute.org/mammals/haploreg/haploreg.php | - |
| **Genenetwork** | Whole blood eQTL data | http://www.genenetwork.org/webqtl/main.py | 61 |
| **GTEx** | eQTL data in other tissue | http://www.gtexportal.org/home | 62 |
| **miRBase** | miRNA information | http://www.mirbase.org | 63 |
| **GeneCards** | A database of human genes | http://www.genecards.org/ | - |

**Table S7. Functional annotation of the 10 identified miRNA binding site variants associated with Alzheimer’s disease**

| **# SNP** | **SNP ID** | **R2** | **EUR** | **GERP** | **SiPhy** | **Prom-Encode** | **Enh-Encode** | **Prom-Roadmap** | **Enh_**  **Roadmap** | **DNAse** | **Protein** | **Motifs** | **Gene ID** | **Loction** | **Proxies** |
| --- | --- | --- | --- | --- | --- | --- | --- | --- | --- | --- | --- | --- | --- | --- | --- |
| 1 | rs6857 | 1 | 0.15 | 1 | 0 | . | . | . | . | . | . | Yes | PVRL2 | U3 | No |
| 2 | rs10119 | 1 | 0.27 | 1 | 0 | . | Yes | . | Yes | . | . | . | TOMM40 | U3 | No |
| 3 | rs28399635 | 1 | 0.19 | 0 | 0 | . | . | . | Yes | Yes | . | Yes | BCAM | U3 | No |
| 4 | rs714948 | 1 | 0.13 | 0 | 0 | . | Yes | . | Yes | Yes | . | . | PVR | U3 | 1 |
|  | rs56261258 | 0.94 | 0.13 | 0 | 0 | Yes | Yes | Yes | Yes | Yes | . | Yes | PVR | INT |  |
| 5 | rs7268 | 1 | 0.55 | 1 | 1 | . | Yes | Yes | Yes | . | . | Yes | HBEGF | U3 | 8 |
|  | rs2878896 | 0.81 | 0.51 | 0 | 0 | . | . | . | . | . | . | Yes | HBEGF | . |  |
|  | rs11168036 | 0.81 | 0.5 | 1 | 1 | . | Yes | Yes | Yes | Yes | Yes | Yes | HBEGF | . |  |
|  | rs6884244 | 0.98 | 0.56 | 0 | 0 | . | Yes | . | Yes | . | . | Yes | HBEGF | . |  |
|  | rs10875633 | 0.98 | 0.56 | 0 | 0 | . | . | . | . | . | . | Yes | HBEGF | . |  |
|  | rs2282802 | 0.99 | 0.55 | 0 | 0 | . | Yes | . | Yes | . | . | Yes | HBEGF | . |  |
|  | rs11168040 | 0.97 | 0.56 | 0 | 0 | . | . | . | . | . | . | Yes | HBEGF | . |  |
|  | rs2074612 | 0.95 | 0.56 | 0 | 0 | . | . | . | Yes | Yes | . | . | HBEGF | INT |  |
|  | rs2074613 | 0.97 | 0.56 | 0 | 0 | . | . | . | Yes | Yes | . | . | HBEGF | INT |  |
| 6 | rs2070736 | 1 | 0.32 | 0 | 0 | . | Yes | Yes | Yes | Yes | Yes | . | DMWD | U3 | 18 |
|  | rs6509237 | 0.81 | 0.66 | 0 | 0 | . | . | . | . | . | . | Yes | SYMPK | INT |  |
|  | rs4802274 | 0.93 | 0.32 | 0 | 0 | . | . | . | . | . | . | Yes | AC074212.3 | INT |  |
|  | rs79987778 | 0.92 | 0.32 | 0 | 0 | . | . | . | . | . | . | Yes | AC074212.3 | INT |  |
|  | rs8106955 | 0.82 | 0.66 | 1 | 1 | . | . | . | Yes | . | . | Yes | SYMPK | INT |  |
|  | rs10412574 | 0.81 | 0.66 | 0 | 0 | Yes | . | Yes | Yes | Yes | Yes | Yes | FOXA3 | . |  |
|  | rs56178951 | 0.81 | 0.34 | 0 | 0 | . | . | . | Yes | . | . | Yes | SYMPK | INT |  |
|  | rs34190352 | 0.99 | 0.32 | 0 | 0 | . | . | . | Yes | . | . | Yes | DMWD | INT |  |
|  | rs4803861 | 0.81 | 0.34 | 0 | 0 | . | . | . | Yes | Yes | . | Yes | SYMPK | INT |  |
|  | rs6509236 | 0.82 | 0.66 | 0 | 0 | . | . | . | . | . | . | Yes | SYMPK | INT |  |
|  | rs16980013 | 0.97 | 0.32 | 0 | 0 | . | Yes | Yes | Yes | Yes | Yes | Yes | AC074212.5 | . |  |
|  | rs4802273 | 0.82 | 0.3 | 0 | 0 | . | . | . | . | . | . | Yes | AC074212.3 | INT |  |
|  | rs4803857 | 0.81 | 0.34 | 0 | 0 | . | . | . | Yes | Yes | . | Yes | SYMPK | INT |  |
|  | rs4514788 | 0.84 | 0.34 | 0 | 0 | . | Yes | Yes | Yes | Yes | . | Yes | RSPH6A | INT |  |
|  | rs10401439 | 0.81 | 0.34 | 0 | 0 | . | Yes | Yes | Yes | . | Yes | . | SYMPK | INT |  |
|  | rs918490 | 0.83 | 0.66 | 0 | 0 | . | . | . | Yes | Yes | . | . | SYMPK | INT |  |
|  | rs55681266 | 0.99 | 0.32 | 0 | 0 | . | . | . | Yes | Yes | . | . | DMWD | INT |  |
|  | rs4286201 | 0.84 | 0.34 | 0 | 0 | . | Yes | . | Yes | . | . | . | RSPH6A | INT |  |
|  | rs73047896 | 0.83 | 0.34 | 0 | 0 | . | . | . | . | . | . | . | SYMPK | INT |  |
| 7,8 | rs1048699 | 1 | 0.1 | 1 | 1 | . | . | . | Yes | Yes | . | Yes | PPP1R37 | U3 | 18 |
| 7,8 | rs74846209 | 0.99 | 0.1 | 0 | 0 | . | . | . | Yes | . | Yes | Yes | PPP1R37 | U3 | 18 |
|  | rs34545713 | 0.94 | 0.11 | 0 | 0 | . | Yes | . | Yes | . | . | Yes | PPP1R37 | INT |  |
|  | rs754366 | 0.94 | 0.11 | 0 | 0 | Yes | Yes | Yes | Yes | Yes | Yes | Yes | PPP1R37 | INT |  |
|  | rs10405086 | 0.94 | 0.11 | 0 | 0 | . | Yes | Yes | Yes | . | . | Yes | PPP1R37 | INT |  |
|  | rs17643262 | 0.94 | 0.11 | 1 | 0 | . | . | . | Yes | . | . | Yes | PPP1R37 | INT |  |
|  | rs113321260 | 0.99 | 0.1 | 0 | 0 | . | . | . | Yes | . | . | Yes | PPP1R37 | . |  |
|  | rs78273125 | 0.94 | 0.11 | 0 | 0 | . | . | . | Yes | Yes | . | Yes | PPP1R37 | INT |  |
|  | rs149151450 | 0.93 | 0.1 | 0 | 0 | Yes | Yes | Yes | Yes | . | . | Yes | PPP1R37 | INT |  |
|  | rs1114832 | 0.93 | 0.11 | 0 | 0 | . | . | . | Yes | . | . | Yes | PPP1R37 | INT |  |
|  | rs10401157 | 0.94 | 0.11 | 0 | 0 | . | Yes | . | Yes | Yes | . | Yes | PPP1R37 | INT |  |
|  | rs144328302 | 0.94 | 0.11 | 0 | 0 | . | Yes | Yes | Yes | . | . | Yes | PPP1R37 | INT |  |
|  | rs143019611 | 0.97 | 0.1 | 0 | 0 | . | . | . | Yes | . | . | Yes | PPP1R37 | . |  |
|  | rs10401823 | 0.94 | 0.11 | 0 | 0 | . | . | . | Yes | Yes | . | Yes | PPP1R37 | INT |  |
|  | rs28469095 | 0.94 | 0.11 | 0 | 0 | Yes | Yes | Yes | Yes | Yes | Yes | Yes | NKPD1 | NSM |  |
|  | rs7248421 | 0.94 | 0.11 | 0 | 0 | . | Yes | Yes | Yes | Yes | . | Yes | PPP1R37 | INT |  |
|  | rs28620490 | 0.94 | 0.11 | 0 | 0 | . | Yes | . | Yes | . | . | Yes | PPP1R37 | INT |  |
|  | rs1114831 | 0.94 | 0.11 | 0 | 0 | . | . | . | Yes | . | . | . | PPP1R37 | INT |  |
|  | rs12462040 | 0.94 | 0.11 | 0 | 0 | . | . | . | Yes | . | . | . | PPP1R37 | INT |  |
|  | rs2004357 | 0.94 | 0.11 | 0 | 0 | . | . | . | Yes | . | . | . | PPP1R37 | INT |  |
| 9 | rs610932 | 1 | 0.56 | 0 | 0 | . | Yes | . | . | . | . | Yes | MS4A6A | U3 | 72 |
|  | rs636147 | 0.93 | 0.55 | 0 | 0 | . | . | . | . | . | . | Yes | MS4A6A | . |  |
|  | rs7946992 | 0.83 | 0.41 | 0 | 0 | . | . | . | . | . | . | Yes | MS4A6A | INT |  |
|  | rs12453 | 0.84 | 0.42 | 0 | 0 | . | . | . | . | Yes | . | Yes | MS4A6A | SYN |  |
|  | rs7935829 | 0.84 | 0.42 | 0 | 0 | . | . | . | Yes | . | . | Yes | MS4A6A | INT |  |
|  | rs631853 | 0.96 | 0.55 | 0 | 0 | . | . | . | . | . | . | Yes | MS4A6A | INT |  |
|  | rs634475 | 0.96 | 0.55 | 0 | 0 | . | . | . | Yes | . | . | Yes | MS4A6A | INT |  |
|  | rs667897 | 0.87 | 0.48 | 0 | 0 | . | Yes | . | Yes | Yes | Yes | Yes | MS4A6A | . |  |
|  | rs624663 | 0.95 | 0.55 | 0 | 0 | . | . | . | . | . | . | Yes | MS4A6A | INT |  |
|  | rs662196 | 0.96 | 0.55 | 0 | 0 | . | . | . | Yes | . | . | Yes | MS4A6A | INT |  |
|  | rs632185 | 0.95 | 0.55 | 0 | 0 | . | Yes | Yes | Yes | Yes | . | Yes | MS4A6A | INT |  |
|  | rs17602572 | 0.83 | 0.41 | 0 | 0 | . | . | Yes | Yes | . | . | Yes | MS4A6A | INT |  |
|  | rs1820430 | 0.81 | 0.42 | 0 | 0 | . | Yes | . | Yes | . | . | Yes | MS4A6A | . |  |
|  | rs2278867 | 0.84 | 0.42 | 0 | 0 | . | Yes | . | Yes | Yes | . | Yes | MS4A6A | INT |  |
|  | rs11230180 | 0.81 | 0.42 | 0 | 0 | . | Yes | . | Yes | . | Yes | Yes | MS4A4E | . |  |
|  | rs56201148 | 0.81 | 0.42 | 0 | 0 | . | . | . | Yes | . | . | Yes | MS4A6A | . |  |
|  | rs1834549 | 0.81 | 0.42 | 0 | 0 | . | . | . | . | . | . | Yes | MS4A6A | . |  |
|  | rs10897011 | 0.81 | 0.42 | 0 | 0 | . | Yes | . | Yes | Yes | Yes | . | MS4A4E | . |  |
|  | rs583791 | 0.95 | 0.55 | 0 | 0 | . | Yes | Yes | Yes | . | . | . | MS4A6A | INT |  |
|  | rs72918674 | 0.84 | 0.42 | 0 | 0 | . | . | . | Yes | . | . | . | MS4A6A | INT |  |
|  | rs2081545 | 0.81 | 0.42 | 0 | 0 | . | . | . | . | . | . | . | MS4A6A | . |  |
| 10 | rs2847655 | 1 | 0.43 | 0 | 0 | . | Yes | . | Yes | . | . | Yes | MS4A2 | U3 | 94 |
|  | rs12226022 | 0.88 | 0.42 | 0 | 0 | . | . | . | Yes | . | . | Yes | AP001257.1 | . |  |
|  | rs11230155 | 0.91 | 0.43 | 0 | 0 | . | . | . | . | . | . | Yes | AP001257.1 | . |  |
|  | rs1813217 | 0.97 | 0.44 | 0 | 0 | . | Yes | . | Yes | . | . | Yes | MS4A2 | . |  |
|  | rs2165525 | 0.91 | 0.43 | 0 | 0 | . | . | . | . | . | . | Yes | AP001257.1 | . |  |
|  | rs107903 | 0.89 | 0.46 | 0 | 0 | . | Yes | . | . | Yes | Yes | Yes | AP001257.1 | . |  |
|  | rs4939314 | 0.91 | 0.43 | 0 | 0 | . | . | . | . | . | . | Yes | AP001257.1 | . |  |
|  | rs2847664 | 0.96 | 0.43 | 0 | 0 | . | . | . | . | . | . | Yes | MS4A2 | INT |  |
|  | rs476722 | 0.89 | 0.46 | 0 | 0 | . | . | . | . | . | . | Yes | AP001257.1 | . |  |
|  | rs11230165 | 0.9 | 0.43 | 0 | 0 | . | . | . | . | . | . | Yes | AP001257.1 | . |  |
|  | rs563803 | 0.87 | 0.46 | 0 | 0 | . | Yes | . | . | . | . | Yes | MS4A2 | . |  |
|  | rs11230147 | 0.95 | 0.44 | 0 | 0 | . | Yes | . | . | . | . | Yes | MS4A2 | . |  |
|  | rs569046 | 0.89 | 0.46 | 0 | 0 | . | . | . | . | . | . | Yes | AP001257.1 | . |  |
|  | rs617135 | 0.87 | 0.47 | 0 | 0 | . | Yes | . | Yes | Yes | Yes | Yes | MS4A6A | . |  |
|  | rs493692 | 0.89 | 0.46 | 0 | 0 | . | . | . | . | . | . | Yes | AP001257.1 | . |  |
|  | rs1303621 | 0.88 | 0.46 | 0 | 0 | . | Yes | . | . | . | . | Yes | MS4A2 | . |  |
|  | rs574695 | 0.87 | 0.46 | 0 | 0 | . | Yes | . | Yes | . | . | Yes | MS4A2 | . |  |
|  | rs583296 | 0.88 | 0.47 | 0 | 0 | . | . | . | . | . | . | Yes | AP001257.1 | . |  |
|  | rs534273 | 0.89 | 0.46 | 0 | 0 | . | . | . | . | . | . | Yes | AP001257.1 | . |  |
|  | rs983392 | 0.93 | 0.42 | 0 | 0 | Yes | Yes | . | Yes | Yes | . | Yes | AP001257.1 | . |  |
|  | rs61901691 | 0.94 | 0.42 | 0 | 0 | . | . | . | . | . | . | Yes | AP001257.1 | . |  |
|  | rs602396 | 0.88 | 0.46 | 0 | 0 | . | Yes | . | . | Yes | Yes | Yes | AP001257.1 | . |  |
|  | rs2855017 | 0.97 | 0.44 | 0 | 0 | . | Yes | Yes | Yes | Yes | Yes | Yes | MS4A2 | . |  |
|  | rs11230161 | 0.91 | 0.43 | 0 | 0 | . | . | . | . | . | . | Yes | AP001257.1 | . |  |
|  | rs7124974 | 0.94 | 0.42 | 0 | 0 | . | . | . | . | . | . | Yes | AP001257.1 | . |  |
|  | rs1303615 | 0.87 | 0.46 | 0 | 0 | . | Yes | . | Yes | Yes | . | Yes | MS4A2 | . |  |
|  | rs55847558 | 0.9 | 0.43 | 0 | 0 | . | . | . | . | . | . | Yes | AP001257.1 | . |  |
|  | rs556917 | 0.84 | 0.47 | 0 | 0 | . | . | . | . | . | . | Yes | MS4A2 | INT |  |
|  | rs556917 | 0.83 | 0.47 | 0 | 0 | . | . | . | . | . | . | Yes | MS4A2 | INT |  |
|  | rs502419 | 0.84 | 0.47 | 0 | 0 | . | Yes | . | Yes | Yes | Yes | Yes | MS4A2 | . |  |
|  | rs502419 | 0.83 | 0.47 | 0 | 0 | . | Yes | . | Yes | Yes | Yes | Yes | MS4A2 | . |  |
|  | rs595481 | 0.89 | 0.46 | 0 | 0 | . | . | . | . | . | . | Yes | AP001257.1 | . |  |
|  | rs9667329 | 0.9 | 0.43 | 0 | 0 | . | . | . | . | . | . | Yes | AP001257.1 | . |  |
|  | rs516478 | 0.83 | 0.47 | 0 | 0 | . | Yes | . | . | . | . | Yes | MS4A2 | . |  |
|  | rs504272 | 0.89 | 0.46 | 0 | 0 | . | . | . | . | . | . | Yes | AP001257.1 | . |  |
|  | rs143391047 | 0.82 | 0.47 | 0 | 0 | . | . | . | . | . | . | Yes | MS4A2 | . |  |
|  | rs143391047 | 0.81 | 0.47 | 0 | 0 | . | . | . | . | . | . | Yes | MS4A2 | . |  |
|  | rs11230160 | 0.91 | 0.43 | 0 | 0 | . | . | . | . | . | . | Yes | AP001257.1 | . |  |
|  | rs2583476 | 0.96 | 0.43 | 0 | 0 | . | . | . | . | . | . | Yes | MS4A2 | INT |  |
|  | rs2070970 | 0.96 | 0.43 | 0 | 0 | . | . | . | Yes | . | . | Yes | MS4A2 | INT |  |
|  | rs7926954 | 0.86 | 0.41 | 0 | 0 | . | Yes | . | . | . | . | Yes | AP001257.1 | . |  |
|  | rs34901701 | 0.92 | 0.43 | 0 | 0 | . | Yes | . | Yes | . | . | Yes | MS4A2 | . |  |
|  | rs510518 | 0.89 | 0.46 | 0 | 0 | . | . | . | . | . | . | Yes | AP001257.1 | . |  |
|  | rs684961 | 0.89 | 0.46 | 0 | 0 | . | . | . | . | . | . | Yes | AP001257.1 | . |  |
|  | rs1125357 | 0.91 | 0.43 | 0 | 0 | . | Yes | . | Yes | . | . | Yes | MS4A2 | . |  |
|  | rs11605427 | 0.86 | 0.41 | 0 | 0 | . | Yes | . | Yes | Yes | Yes | Yes | AP001257.1 | . |  |
|  | rs4939311 | 0.93 | 0.43 | 0 | 0 | . | Yes | . | . | . | . | Yes | MS4A2 | . |  |
|  | rs138369826 | 0.9 | 0.43 | 0 | 0 | . | . | . | . | . | . | Yes | AP001257.1 | . |  |
|  | rs581133 | 0.87 | 0.46 | 0 | 0 | . | . | . | Yes | . | . | Yes | MS4A2 | . |  |
|  | rs487997 | 0.84 | 0.47 | 0 | 0 | . | Yes | . | Yes | . | . | Yes | MS4A2 | . |  |
|  | rs487997 | 0.83 | 0.47 | 0 | 0 | . | Yes | . | Yes | . | . | Yes | MS4A2 | . |  |
|  | rs11600716 | 0.91 | 0.43 | 0 | 0 | . | . | . | . | . | . | Yes | AP001257.1 | . |  |
|  | rs200623768 | 0.81 | 0.44 | 0 | 0 | . | . | . | . | . | . | Yes | AP001257.1 | . |  |
|  | rs35408863 | 0.86 | 0.46 | 0 | 0 | . | . | . | . | . | . | Yes | AP001257.1 | . |  |
|  | rs7933202 | 0.83 | 0.4 | 0 | 0 | . | Yes | . | Yes | Yes | Yes | Yes | MS4A6A | . |  |
|  | rs7933202 | 0.84 | 0.4 | 0 | 0 | . | Yes | . | Yes | Yes | Yes | Yes | MS4A6A | . |  |
|  | rs688030 | 0.89 | 0.46 | 0 | 0 | . | . | . | . | . | . | Yes | AP001257.1 | . |  |
|  | rs606588 | 0.89 | 0.46 | 0 | 0 | . | . | . | . | . | . | Yes | AP001257.1 | . |  |
|  | rs558375 | 0.89 | 0.46 | 0 | 0 | . | . | . | . | . | . | Yes | AP001257.1 | . |  |
|  | rs1786137 | 0.83 | 0.47 | 0 | 0 | . | . | . | . | Yes | . | Yes | MS4A2 | . |  |
|  | rs1786137 | 0.82 | 0.47 | 0 | 0 | . | . | . | . | Yes | . | Yes | MS4A2 | . |  |
|  | rs514266 | 0.87 | 0.46 | 0 | 0 | . | Yes | . | . | . | . | Yes | MS4A2 | . |  |
|  | rs17529983 | 0.89 | 0.43 | 0 | 0 | . | Yes | . | . | Yes | . | Yes | AP001257.1 | . |  |
|  | rs10897009 | 0.91 | 0.43 | 0 | 0 | . | . | . | Yes | . | . | Yes | AP001257.1 | . |  |
|  | rs4939312 | 0.91 | 0.43 | 0 | 0 | . | . | . | . | . | . | Yes | AP001257.1 | . |  |
|  | rs652354 | 0.89 | 0.46 | 0 | 0 | . | Yes | Yes | Yes | . | . | Yes | AP001257.1 | . |  |
|  | rs2847663 | 0.96 | 0.43 | 0 | 0 | . | . | . | . | . | . | Yes | MS4A2 | INT |  |
|  | rs540170 | 0.87 | 0.46 | 0 | 0 | . | Yes | . | Yes | . | . | Yes | MS4A2 | . |  |
|  | rs555635 | 0.81 | 0.47 | 0 | 0 | . | Yes | . | Yes | . | . | Yes | MS4A2 | . |  |
|  | rs555635 | 0.86 | 0.47 | 0 | 0 | . | Yes | . | Yes | . | . | Yes | MS4A2 | . |  |
|  | rs580817 | 0.84 | 0.47 | 0 | 0 | . | Yes | Yes | Yes | Yes | . | Yes | MS4A2 | . |  |
|  | rs580817 | 0.83 | 0.47 | 0 | 0 | . | Yes | Yes | Yes | Yes | . | Yes | MS4A2 | . |  |
|  | rs574704 | 0.84 | 0.47 | 0 | 0 | . | Yes | Yes | Yes | . | . | Yes | MS4A2 | . |  |
|  | rs574704 | 0.83 | 0.47 | 0 | 0 | . | Yes | Yes | Yes | . | . | Yes | MS4A2 | . |  |
|  | rs2847668 | 0.96 | 0.43 | 0 | 0 | . | . | . | . | . | . | Yes | MS4A2 | INT |  |
|  | rs483629 | 0.89 | 0.46 | 0 | 0 | . | . | . | Yes | . | . | Yes | AP001257.1 | . |  |
|  | rs1441586 | 0.82 | 0.46 | 0 | 0 | . | Yes | . | . | . | . | Yes | MS4A2 | . |  |
|  | rs1863472 | 0.91 | 0.43 | 0 | 0 | . | . | . | . | . | . | Yes | AP001257.1 | . |  |
|  | rs11230153 | 0.91 | 0.43 | 0 | 0 | . | . | . | Yes | . | . | Yes | AP001257.1 | . |  |
|  | rs512495 | 0.82 | 0.47 | 0 | 0 | . | Yes | Yes | Yes | Yes | . | Yes | MS4A2 | . |  |
|  | rs512495 | 0.85 | 0.47 | 0 | 0 | . | Yes | Yes | Yes | Yes | . | Yes | MS4A2 | . |  |
|  | rs564912 | 0.88 | 0.46 | 0 | 0 | . | . | . | Yes | Yes | Yes | . | MS4A2 | . |  |
|  | rs525794 | 0.89 | 0.46 | 0 | 0 | . | . | . | . | Yes | . | . | AP001257.1 | . |  |
|  | rs574798 | 0.87 | 0.46 | 0 | 0 | . | Yes | . | Yes | . | . | . | MS4A2 | . |  |
|  | rs17528859 | 0.97 | 0.44 | 0 | 0 | . | Yes | . | Yes | . | . | . | MS4A2 | . |  |
|  | rs2583471 | 0.96 | 0.43 | 0 | 0 | . | . | . | Yes | . | . | . | MS4A2 | INT |  |
|  | rs521952 | 0.85 | 0.47 | 0 | 0 | . | Yes | . | Yes | . | . | . | MS4A2 | . |  |
|  | rs521952 | 0.82 | 0.47 | 0 | 0 | . | Yes | . | Yes | . | . | . | MS4A2 | . |  |
|  | rs502581 | 0.84 | 0.47 | 0 | 0 | . | Yes | . | . | . | . | . | MS4A2 | INT |  |
|  | rs502581 | 0.83 | 0.47 | 0 | 0 | . | Yes | . | . | . | . | . | MS4A2 | INT |  |
|  | rs2847666 | 0.99 | 0.42 | 0 | 0 | . | . | . | . | . | . | . | MS4A2 | INT |  |
|  | rs2847667 | 0.99 | 0.42 | 0 | 0 | . | . | . | . | . | . | . | MS4A2 | INT |  |
|  | rs920573 | 0.91 | 0.43 | 0 | 0 | . | . | . | . | . | . | . | AP001257.1 | . |  |
|  | rs1316005 | 0.89 | 0.46 | 0 | 0 | . | . | . | . | . | . | . | AP001257.1 | . |  |
|  | rs617916 | 0.89 | 0.46 | 0 | 0 | . | . | . | . | . | . | . | AP001257.1 | . |  |

In this table each of the 10 miRNA binding site-SNPs have shown with different colors. R2, LD correlation; EUR, European ancestry; GERP and SiPhy, Conservation; Prom, Promoter; Enh, Enhancer; Proxies, Number of proxy SNPs; Yes, existing data; U3, 3’UTR; U5, 5”UTR; INT, Intron; NSM, Non-synonyms.

**Table S8. List o the primers for the cloning of the miR-1229 precursor containing wild-type and mutant alleles**

| **miRNA primer** | **Sequence** |
| --- | --- |
| **miR-1229**  **Wt Fw** | tcgagGATCGGTGGGTAGGGTTTGGGGGAGAGCGTGGGCTGGGGTTCAGGGACA  CCCTCTCACCACTGCCCTCCCACAGGCTCCg |
| **miR-1229**  **Wt Rev** | aattcGGAGCCTGTGGGAGGGCAGTGGTGAGAGGGTGTCCCTGAACCCCAGCCCA  CGCTCTCCCCCAAACCCTACCCACCGATCc |
| **miR-1229 Mut Fw** | tcgagGATCGGTGGGTAGGGTTTGGGGGAGAGTGTGGGCTGGGGTTCAGGGACA  CCCTCTCACCACTGCCCTCCCACAGGCTCCg |
| **miR-1229 Mut Rev** | aattcGGAGCCTGTGGGAGGGCAGTGGTGAGAGGGTGTCCCTGAACCCCAGCCCA  CACTCTCCCCCAAACCCTACCCACCGATCc |

Wt, wild-type; Mut, Mutation; Fw, Forward; Rev, Reverse.

**Table S9. List of the primers for the cloning of the wild type and mutant 3’UTR of SORL1**

| **primer** | **Sequence** |
| --- | --- |
| **SORL1 Wt FW** | 5’ CTAGAGAGGCCATGTCTGTGCAGTCCTAGTTCCAGACAGGTGAGAAGCTCCA  GGAACTACTGGCTACCTTGACAAGGGCC 3’ |
| **SORL1 Wt RV** | 5’ CTTGTCAAGGTAGCCAGTAGTTCCTGGAGCTTCTCACCTGTCTGGAACTAGGA  CTGCACAGACATGGCCTCT 3’ |
| **SORL1 Mut FW** | 5’ CTAGAGAGGCCATGTCTGTGCAGTCCTAGTTCCAGACAGTTGATAAGCTCCA  GGAACTACTGGCTACCTTGACAAGGGCC 3’ |
| **SORL1 Mut RV** | 5’ CTTGTCAAGGTAGCCAGTAGTTCCTGGAGCTTATCAACTGTCTGGAACTAGGA  CTGCACAGACATGGCCTCT 3’ |

Wt, wild-type; Mut, Mutation; Fw, Forward; Rev, Reverse.

**Figure S1. Regional plot showing the association of miR-1229 variant with Alzheimer’s disease**

**
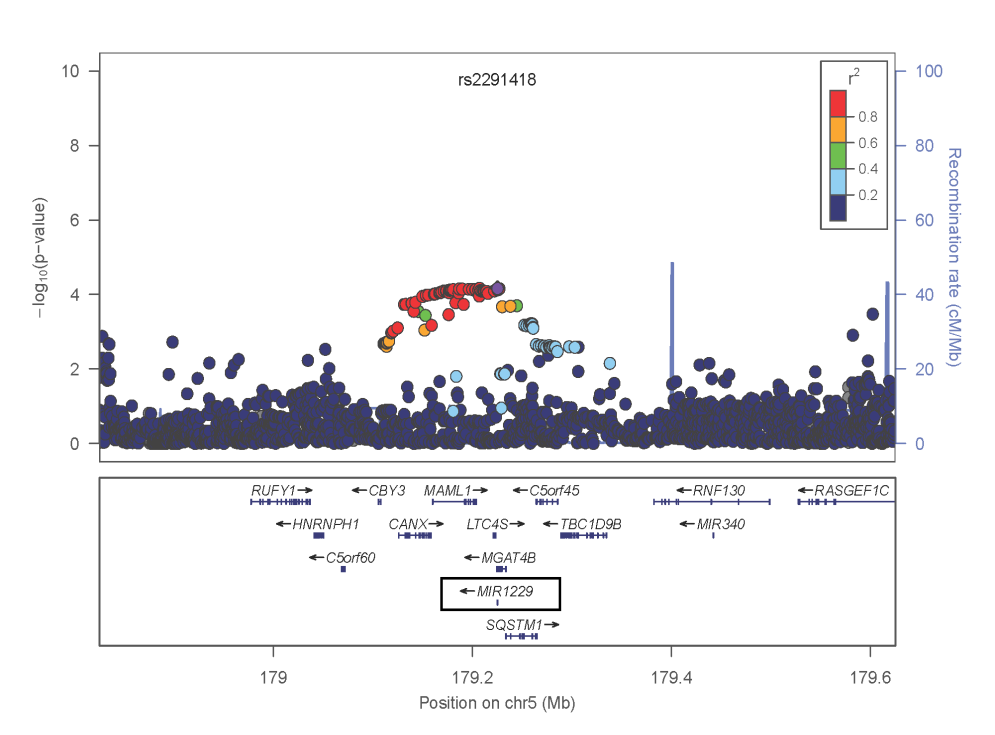
**

**Figure S2. A dose-dependent regulation of *SORL1* expression by miR-1229-3p**


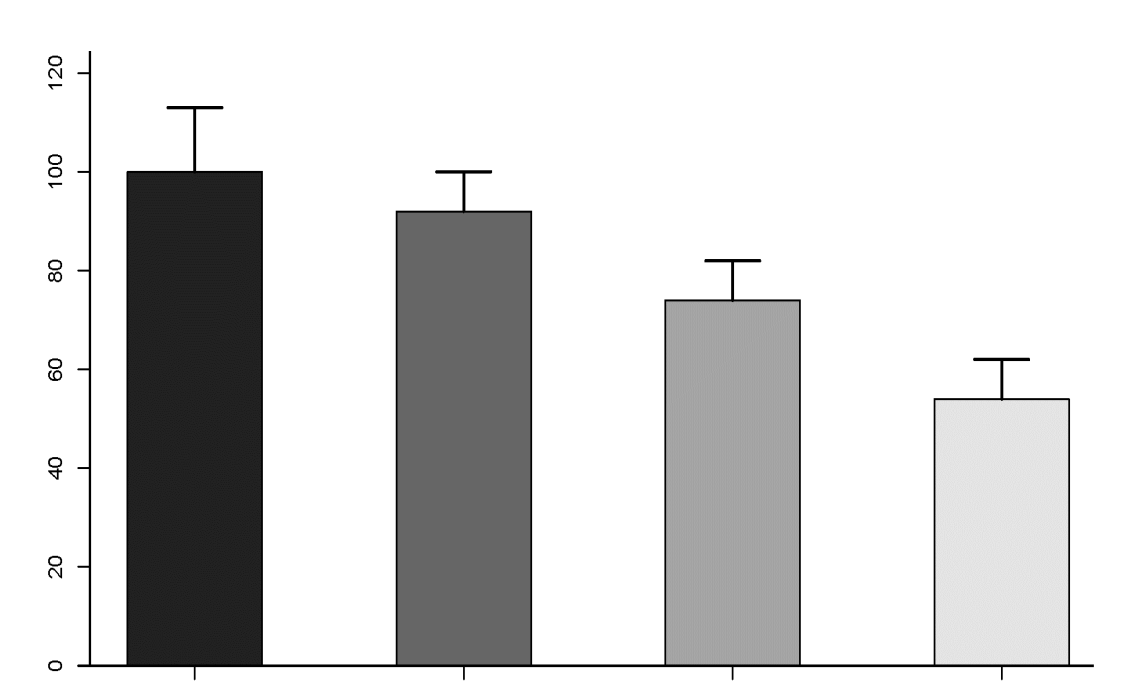


**Luciferase reporter activity of SORL1 (%)**

***SORL1* (wt) +**

**miR-1229-3p (10 ng)**

***SORL1* (wt) +**

**miR-1229-3p (50 ng)**

***SORL1* (wt) +**

**miR-1229-3p (20 ng)**

***SORL1*(wt) +**

**miR-1229-3p (30 ng)**

*

*

This figure shows luciferase reporter assays for *SORL1* gene with four different concentrations of miR-1229-3p . We have more reduction in the relative luciferase activity of wild type *SORL1* reporter by increasing the concentration of miR-1229-3p.

**Figure S3. Regional association plots of the identified miRNA-binding site variants associated with Alzheimer’s disease**

**
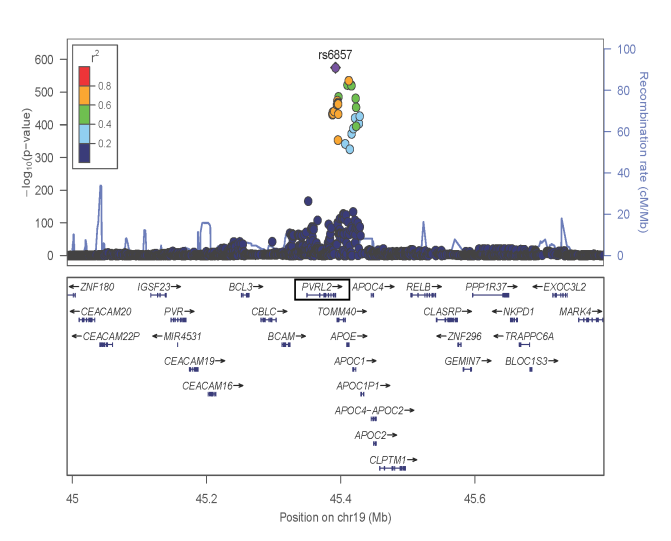

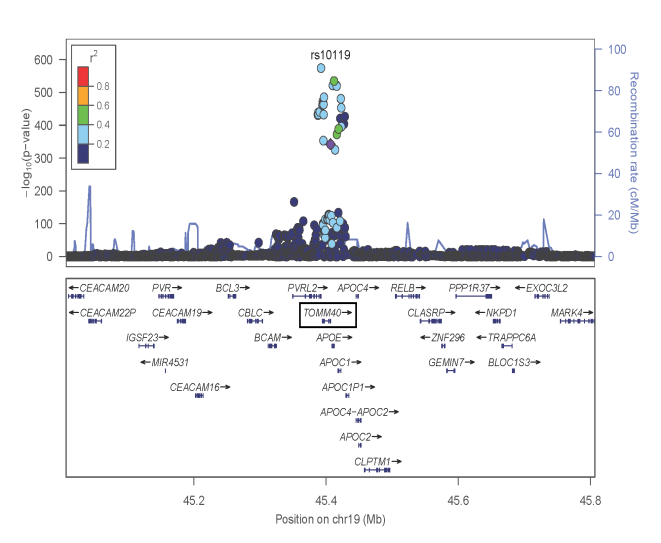

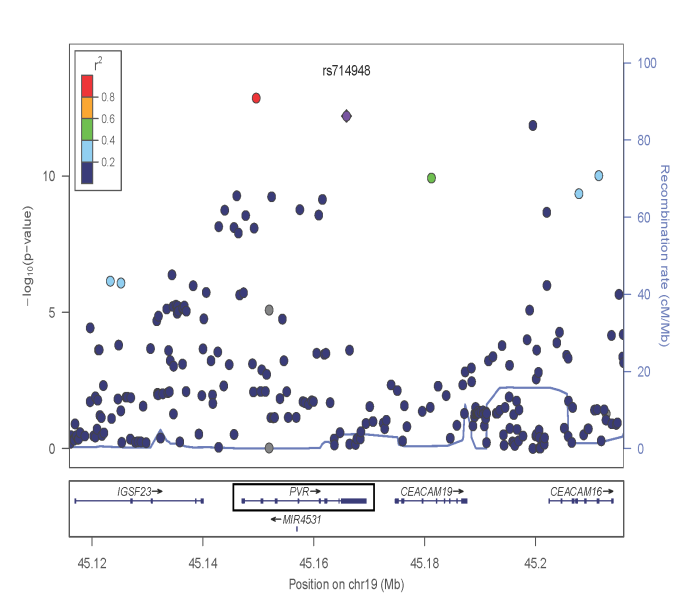
**

**
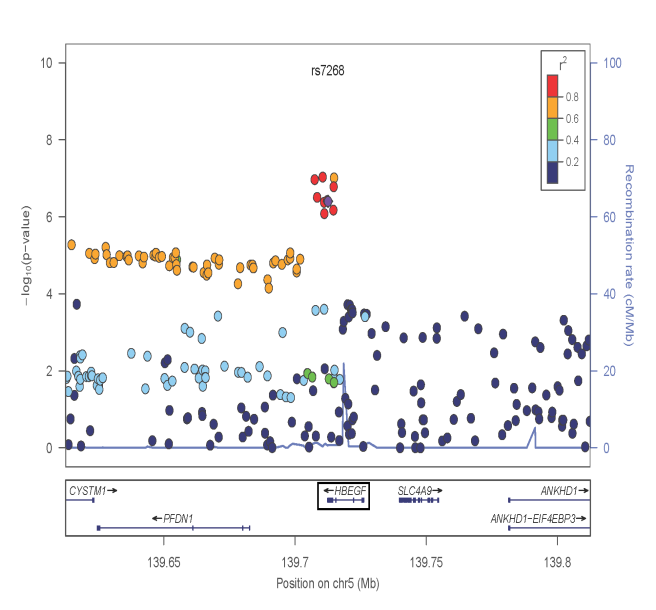

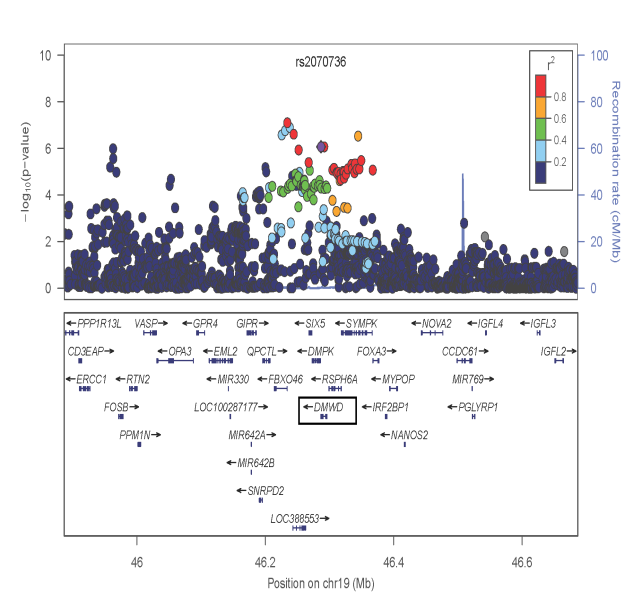

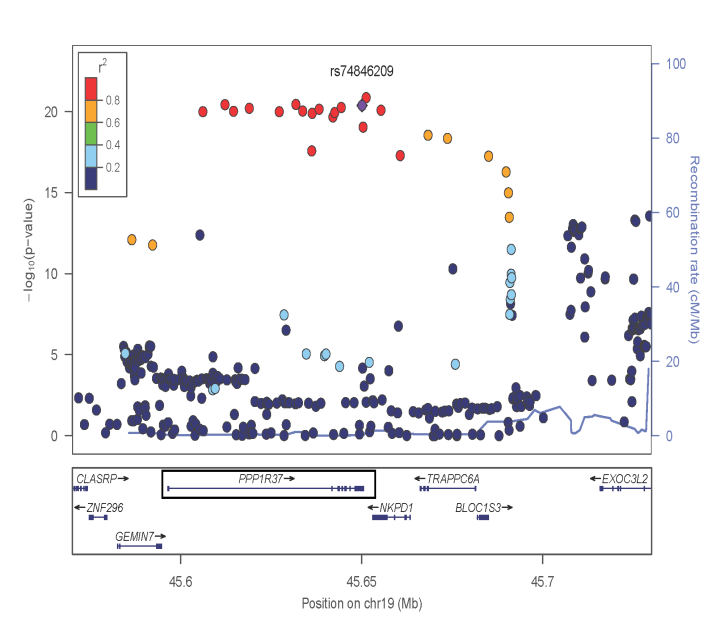
**
